# Effectiveness of Print Education at Reducing Urban Mosquito Infestation through Improved Resident-Based Management

**DOI:** 10.1371/journal.pone.0155011

**Published:** 2016-05-12

**Authors:** Danielle Bodner, Shannon L. LaDeau, Dawn Biehler, Nicole Kirchoff, Paul T. Leisnham

**Affiliations:** 1 Ecosystem Health and Natural Resource Management, Department of Environmental Science and Technology, University of Maryland, College Park, Maryland, United States of America; 2 Cary Institute of Ecosystem Studies, Millbrook, New York, United States of America; 3 Geography and Environmental Systems, University of Maryland, Baltimore County, Baltimore, Maryland, United States of America; New Mexico State University, UNITED STATES

## Abstract

Improving resident-based management and knowledge of mosquitoes is often an integral component of integrated mosquito management, especially in urban landscapes with considerable mosquito habitat on privately owned lands. This study tested the effectiveness of print education materials at reducing urban mosquito exposure through improving resident knowledge of, and attitudes towards, mosquitoes and mosquito management in Washington DC, USA. There was a specific focus on the removal of water-filled containers that are utilized by the developmental stages of the two most common vector species in the region, *Aedes albopictus* and *Culex pipiens*. Households in six neighborhoods that varied in socio-economic status were administered knowledge, attitude, and practice (KAP) surveys in 2010 and 2012, and had their yards surveyed for container habitats and immature mosquitoes (larvae and pupae) in 2010, 2011, and 2012. Half the households (intervention, n = 120) received education materials in 2011 and 2012 to yield a before-after control-intervention (BACI) design. Unexpectedly, residents in intervention households were more likely to show decreased concern for mosquito-borne illnesses than residents in control households, which did not receive materials. Moreover, there was a greater probability that control households reduced containers in 2012 than intervention households, particularly when they had low numbers of baseline (2010) containers. Irrespective of control, reductions in containers were associated with decreased abundances of immature mosquitoes. Overall, our findings suggest that print education materials may have unintended negative effects on resident attitudes and household management of mosquito production. We recommend that mosquito control agencies need to carefully consider their content of print messages and the effectiveness of strategies that passively convey information with little or no engagement with control professionals.

## Introduction

Adult female mosquitoes acquire proteins required for egg development by biting humans and other animals, and it is this behavior that makes them important medical and veterinary pests and disease-vectors. Mosquito-borne diseases have important ecological, economic, and human health implications world-wide. For example, there were an estimated 214 million cases of malaria in 2014 [1]. Malaria sustains cycles of morbidity and poverty across generations, and creates a total global economic burden that was estimated to exceed $2.7 billion in 2014 [2]. Similarly, more than one-third of the world’s population is at risk for infection and associated negative social impacts of dengue [3]. In the United States, West Nile virus is the most important mosquito-borne disease, having caused 41,000 diagnosed cases and 1,700 deaths since its first detection in North America in 1999 [4].

The Asian tiger mosquito, *Aedes albopictus* Skuse and the northern house mosquito, *Culex pipiens* L. are among the most important disease-vector mosquito species in North America. *Aedes albopictus* and *Cx*. *pipiens* commonly utilize artificial water-filled containers (e.g., tires, buckets, fence posts, birdbaths) to complete their developmental life-stages (egg, larvae, pupae). *Aedes albopictus* invaded the continental United States in the mid-1980s and has since spread rapidly throughout the eastern part of the country [5–6] to become one of the most common human-biting urban mosquitoes in its new range [7–8]. *Aedes albopictus* is a competent laboratory vector for West Nile virus, La Crosse encephalitis virus, and Eastern equine encephalitis virus [9–11], as well as dengue and chikungunya viruses, which threaten to establish in North America [12–15]. *Culex pipiens* invaded North America over 200 years ago and is common in urban areas throughout the northern United States [16–17]. Although *Cx*. *pipiens* does not typically feed on humans, laboratory and field studies implicate *Cx*. *pipiens* as the principal West Nile virus vector in its range [10, 18].

Urban mosquitoes, including *Ae*. *albopictus* and *Cx*. *pipiens*, are often not amenable to chemical treatments by mosquito professionals. Adulticiding sometimes raises health concerns among resident communities, and is increasingly costly for fiscally-constrained mosquito-control agencies [19]. It is also often ineffective against *Ae*. *albopictus*, which is active during the daytime when spraying is less commonly performed [11]. Urban areas are fragmented into numerous privately owned parcels that can conceal abundant containers and make them inaccessible, thus limiting wide-spread larviciding of important habitats that produce large numbers of adults [19].

Given the constraints of traditional mosquito control, resident-based management of water-filled containers (i.e., source reduction) can be an effective and affordable means of decreasing numbers of biting adult mosquitoes [20], and it is recommended by the World Health Organization for control of urban vector species worldwide [2]. Effective resident-based management of urban mosquitoes requires residents to be knowledgeable and motivated to implement source reduction practices. Public education and outreach are routinely employed by mosquito control and health departments to improve human behaviors and manage local mosquito populations, but evidence for their effectiveness is mixed and confusing (e.g., [21–30]).

The efficacy of education campaigns on improving resident knowledge, reducing mosquito habitats, and controlling adult mosquito production likely involves complex relationships between the demographics of residents and their existing knowledge of, and attitudes towards, mosquitoes. Socioeconomic status (SES) indicators have been associated with different levels of knowledge [31], mosquito control attitudes [32], source reduction, mosquito infestation, and disease incidence (e.g., [33–38]). Previous studies have demonstrated that differences in container volume, purpose, and permanence can influence larval abundance and adult emergence across economically and culturally distinct urban neighborhoods in the mid-Atlantic region of the United States [32, 39], and among differing levels of infrastructural decay in cities [40]. No studies that we are aware of have rigorously addressed these relationships when assessing the effectiveness on education campaigns. Understanding how education interventions may interact with these factors would provide important insights into social and ecological mechanisms driving resident-based mosquito management, as well as help develop a better indication of what instruction approaches are effective.

We evaluated the effectiveness of standard print education materials at reducing abundances of immature *Ae*. *albopictus* and *Cx*. *pipiens* in household yards across socio-economically diverse neighborhoods in Washington DC, USA. We compared improvements in the knowledge, attitudes, and practices (KAP) of residents to mosquitoes over a three-year period between intervention households, which received education materials, versus control households, which did not receive any materials. This study builds on a prior study by Dowling et al. (2013) that found source reduction to be related to overall respondent knowledge of mosquitoes and specific knowledge of mosquito development; both of which varied with specific demographic factors and respondent motivation to control mosquitoes [31]. Specifically, respondents from high SES households reported greater knowledge but lower motivation than respondents from middle and low SES households [31]. The study here directly builds on Dowling et al. (2013) [31] by resampling the same households to re-evaluate KAP responses and test for changes in numbers of water-holding containers and mosquito densities in two additional years (2011 and 2012).

## Methods

### Study sites and education materials

Our study employed a before-after control-intervention (BACI) design to evaluate changes in resident responses and household mosquito infestation following a print education intervention. In the summers of 2011 and 2012, 40 households were resampled in each of five neighborhoods in Washington D.C. (Deanwood, Georgetown, Petworth, Shepherd Park, Trinidad) and one neighborhood in Montgomery Co., Maryland (Silver Spring) that were sampled by Dowling et al. in 2010 (240 total households) [31]. At the beginning of the mosquito season (May) in 2011 and 2012, printed color education materials (S1 Fig) were distributed to 20 randomly selected households (intervention households) in each neighborhood (120 total). Education materials included a calendar, a notepad, a flyer and a magnet with pictorial and written mosquito education information (S1 Fig). The materials in this study were consistent with materials that are commonly distributed by mosquito control agencies (e.g., www.mosquito.org). Materials were mailed to intervention households in 2011. In 2012, the same materials were hand-delivered to intervention households by an investigator. The deployment of materials in May was set to mirror the timing of common mosquito control outreach that intends to educate residents in the early summer so that their behaviors may be influenced over the entire summer when mosquitoes are most active.

Baseline resident knowledge, attitude, and practices (KAP), and mosquito infestation, were assessed in 2010 by administering KAP questionnaires and conducting comprehensive immature mosquito surveys [31]. From June to August in 2011 and 2012, households were re-revisited. Revisits occurred during the same week of the year as initial visits (in 2010) to minimize confounding any changes in KAP or mosquito infestation with time of year. A total of 211 and 158 households were resampled in 2011 and 2012, respectively. A household was not resampled if a resident was not home after five visits, if residents had moved, or if they did not consent to remain in the study. In 2010 and 2012, demographic information was collected on the age, gender, and education of individual respondents, and the income, size (number of residents), and ownership status (rent, own) of respondent households. Human subjects approval for work in 2010 was obtained from the Georgetown University Institutional Review Board (Protocol # 425–2009) [31]. Human subjects approval for work in 2012 was obtained from the University of Maryland, College Park Institutional Review Board (Protocol # 11–0192). In both years, oral consent was obtained from participants after investigators read a verbal script of the research and provided a copy. Oral rather than written consent was obtained because: 1) the research presented no more than minimal risk of harm to subjects; 2) the research involved no procedures for which written consent is normally required outside the research context; and 3) oral communication was deemed easier for participants to understand the study and to provide consent. The oral consent procedure was approved by both the Georgetown University Institutional Review Board (Protocol # 425–2009) and University of Maryland, College Park Institutional Review Board (Protocol # 11–0192). All data was analyzed anonymously.

### Individual-Level Changes in KAP

A total of 107 questionnaires were administered to the same respondents in both 2010 and 2012, allowing the assessment of changes in knowledge, attitudes, and self-reported source reduction of individual residents. Respondents were assigned an overall knowledge score ranging from 0–3 based on their answers to three questions about mosquito ecology and associated diseases [31]. Two questions, concerning respondent attitudes towards mosquito control and motivation to undertake mosquito management, were used in this study. For the first attitude question, respondents rated their concern of diseases transmitted by mosquitoes on a five-point scale. The second attitude question asked residents to identify mosquito control responsibility. Respondents were scored according to whether or not they identified individual residents as having either sole or shared responsibility with control agencies vs. no responsibility for mosquito control. To measure source reduction practices, we asked respondents a yes/no question about whether they reduced mosquito populations in their yard. If an individual respondent reported that they reduced mosquitoes, we then asked that individual what mosquito-reduction strategies they implemented and recorded whether or not they practiced source reduction (e.g., emptying water-holding containers, applying larvicide to immoveable water sources [31]).

A primary goal was to test whether or not passive education materials resulted in improved KAP scores. Thus, changes in individual KAP responses between 2010 and 2012 were subsequently coded as binary variables, with improvements in KAP being scored as those that showed an increased knowledge score over their baseline score, an increase in their degree of concern of mosquito diseases from 1–3 to 4–5, identification of sole or part resident responsibility to reduce mosquitoes after previously not identifying such responsibility, and the adoption of source reduction after previously not doing source reduction (S1 Table). Decreasing or identical scores from the 2010 and 2012 questionnaires indicated no improvement in overall knowledge, no increased degree of concern of mosquito diseases, no increased sense of resident responsibility to reduce mosquitoes, and no adoption of source reduction adoption. Residents that had the highest possible baseline scores for knowledge, a high degree of concern, indicated resident responsibility, or indicated source reduction practice in 2010 were not included in analyses since improvement was not possible.

### Household-level Changes in Source Reduction and Mosquito Infestation

Investigators quantified potential habitat and immature mosquito infestation in each year at all yards (i.e., three times, from 2010 to 2012). During yard surveys, two to three investigators systematically searched for and enumerated all water-holding containers. Container habitats were classified into one of three types (structural, functional, disused/trash) using the same definitions as Dowling et al. [31]. Structural containers were permanent or immoveable artificial containers (e.g. basement drains, gutters, fence posts). Functional containers consisted of moveable and useful containers used for yard care, storage, and recreation (e.g. garbage cans, watering cans, buckets). Disused artificial containers were designated by investigators to be trash (e.g. dumped tires, plastic cups). For each container, water was homogenized and up to 1-L was sampled after the total volume of the container water was recorded. Mosquitoes were isolated from water samples and stored in ethanol for later processing. All mosquitoes in each sample were enumerated, and up to 50 3^rd^ and 4^th^ (late) instar larvae were identified to species [17]. Up to 50 1^st^ and 2^nd^ (early) instar larvae were identified to genus and their species were estimated on species proportions among larvae in the genus that were collected from the same container sample. While larval keys of North American mosquitoes are written to identify mature 4^th^ instar larvae, identification of early instar larvae of some species is possible, and was reliably identified to genus in this study. All pupae from each sample were identified to genus using a pupal key [40, 41], and then also categorized into species based on co-occurring late-instar larvae. Mosquito abundances in each container were estimated (total and by instar and species) by multiplying total container volume by the sampled density. To test potential effects of investigator visits on resident behaviors, ten households that had not previously been visited (double control) were randomly surveyed for total and mosquito-positive containers in Shepherd Park (2011) and Silver Spring (2012) neighborhoods.

### Data Analysis

**Fig 1:**
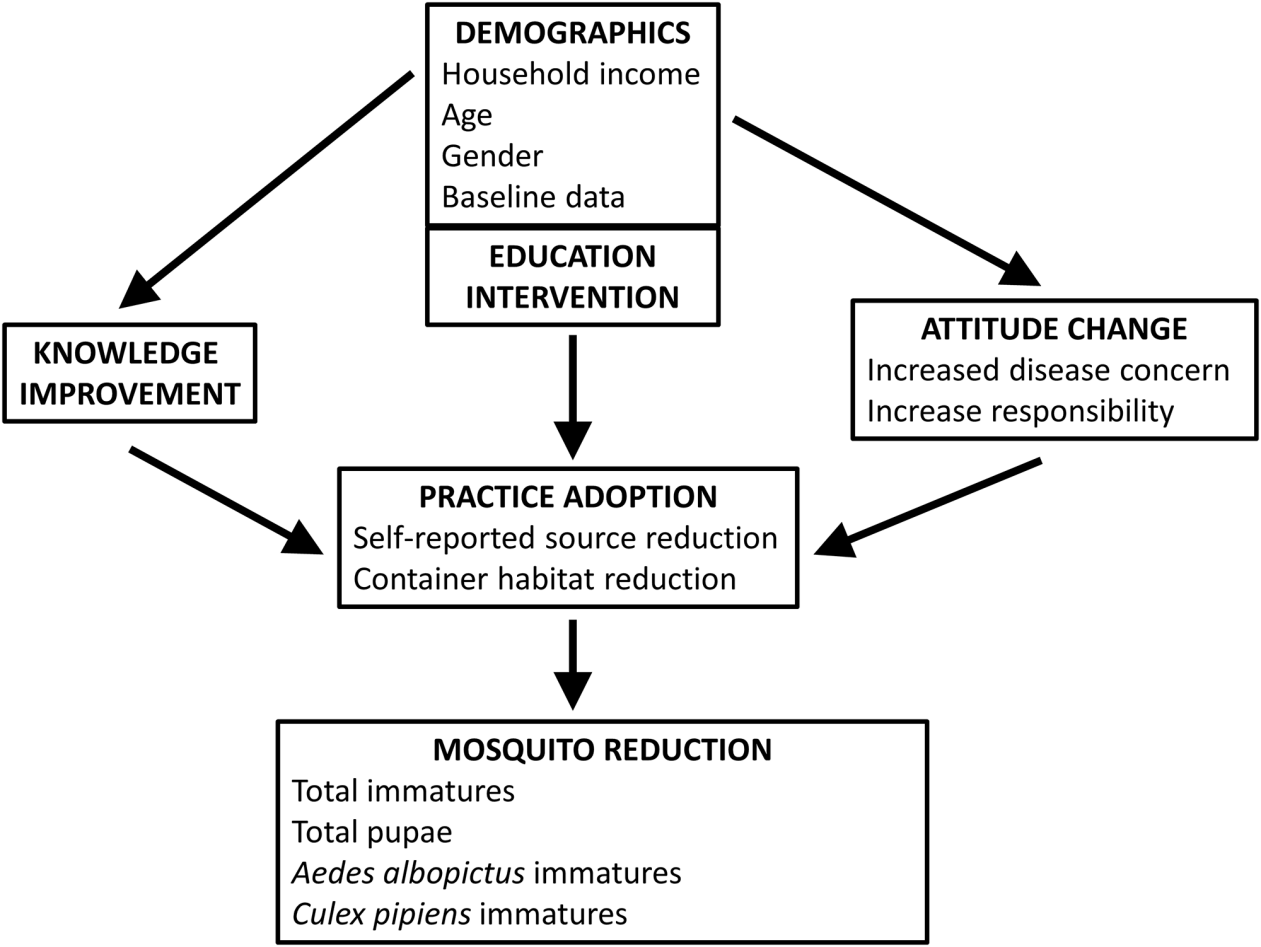
Diagram of Analyses. Relationships among individual demographics, knowledge improvements, attitude change, adoption of source reduction practices, and mosquito reductions that were tested using logistic regressions.

We used logistic regression models to test for differences in individual knowledge improvement, increased concern, increased responsibility, and the adoption of self-reported source reduction between respondents from households that received education materials vs. control households (Fig 1). Our models included demographic variables (household income, age, or gender) that were shown to be important predictors of baseline (2010) KAP responses [31] (Table 1). Logistic regression models were also used to test for a relationship between education intervention and household-level decreases in the abundance of container habitat. Neighborhood and sampling week were included in the model because they have been shown to influence the abundance of immature mosquitoes and backyard container habitats [31]. Two-way interactions with education intervention were included in initial multi-factor models, but these were removed from subsequent tests because they were non-significant. Multi-collinearity was tested for all multifactor models by means of variance inflation characteristics (VIF), with a VIF above 5 for a variable indicating a problem [41]; however, none were evident. Total containers in control and experimental households in Shepherd Park (2011) and Silver Spring (2012) were compared with double control households that had not previously been visited using analysis of variance (ANOVA) to test for any effects of prior investigator visit on container reduction. Associations between household container reduction and reductions in mosquito abundances (i.e., total immatures [larvae + pupae], total pupae, *Ae*. *albopictus* larvae, *Ae*. *albopictus* pupae, *Cx*. *pipiens* larvae, *Cx*. *pipiens* pupae) were tested using Fisher’s Exact Tests. Odd’s ratios (OR) are provided for significant variables to demonstrate the relative strength of the relationship, such that higher OR indicates a greater likelihood of occurrence. Because of the relatively low numbers of individual respondents that were sampled in 2010 and 2012 and because our emphasis was on detecting broad social patterns, we accepted experimentwise α = 0.10 for all tests All statistical summaries and analyses were computed using the R Statistical Software (Version 3.0.2).

**Table 1:** Logistic regression results testing relationships between education intervention and improvements in total knowledge, increased resident responsibility, increased concern, and the self-reported adoption of source reduction.

	df	X^2^	p
**Knowledge Improvement**			
Education	1, 71	0.27	0.606
Age	1, 71	0.69	0.406
Household Income	2, 71	4.21	0.122
Baseline Knowledge	1, 71	3.25	**0.073**
**Increased Concern**			
Education	1, 41	4.07	**0.044**
Gender	1, 41	1.26	0.532
Household Income	2, 41	1.07	0.302
Baseline Attitude	1, 41	0.11	0.745
**Resident Responsibility**			
Education	1, 29	0.02	0.886
Gender	1, 29	0.78	0.677
Household Income	2, 29	0.12	0.726
Baseline Attitude	1, 29	0.93	0.335
**Source Reduction Adoption**			
Education	1, 54	4.00	**0.045**
Age	1, 54	2.26	0.132
Household Income	2, 54	2.63	0.268
Week Sampled	1, 54	0.84	0.358
Knowledge	1, 54	8.23	**0.004**
Responsibility	1, 54	0.42	0.514
Concern	1, 54	0.58	0.445

Baseline responses and important demographic variables from a 2010 questionnaire responses are included in all models.

Significant effects at experimentwise α = 0.10 are shown in bold.

## Results

### Individual-level KAP questionnaire responses

Residents with higher baseline (2010) total knowledge scores were more likely to show improvement in total knowledge (OR = 2.56; Table 1), regardless of education intervention. Increased concern of mosquito-borne diseases was predicted by education intervention (Table 1 and S1 Table), but respondents from control households were more likely to report increased concern than respondents that received education materials (OR = 6.17). Increases in source reduction adoption were independently predicted by education intervention and improvements in total knowledge (Table 1). Residents that received passive education materials (OR = 5.13) and residents with increased total knowledge (OR = 15.99) were more likely to report source reduction adoption than residents in control households and who did not exhibit increased knowledge. Increases in resident-identified responsibility to control mosquitoes was not significantly predicted by any of the variables tested (Table 1), likely because the vast majority of individuals already reported personal responsibility (66/107) in 2010.

### Household-level Changes in Source Reduction and Mosquito Infestation

**Fig 2:**
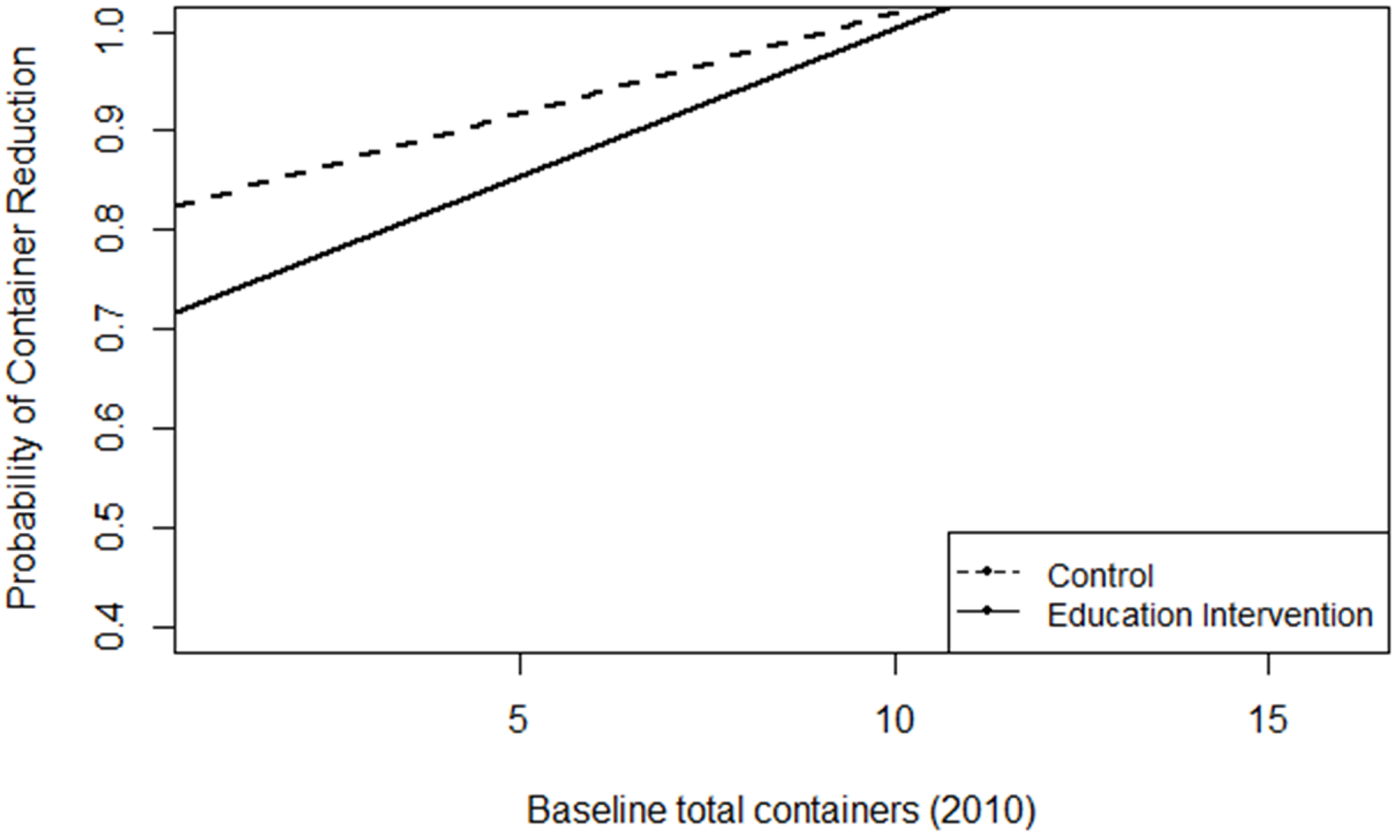
Greater container reductions in control households. Relationship between education intervention and the probability of container reduction from 2010 to 2012 across households with different numbers of baseline (2010) water-holding containers.

**Fig 3:**
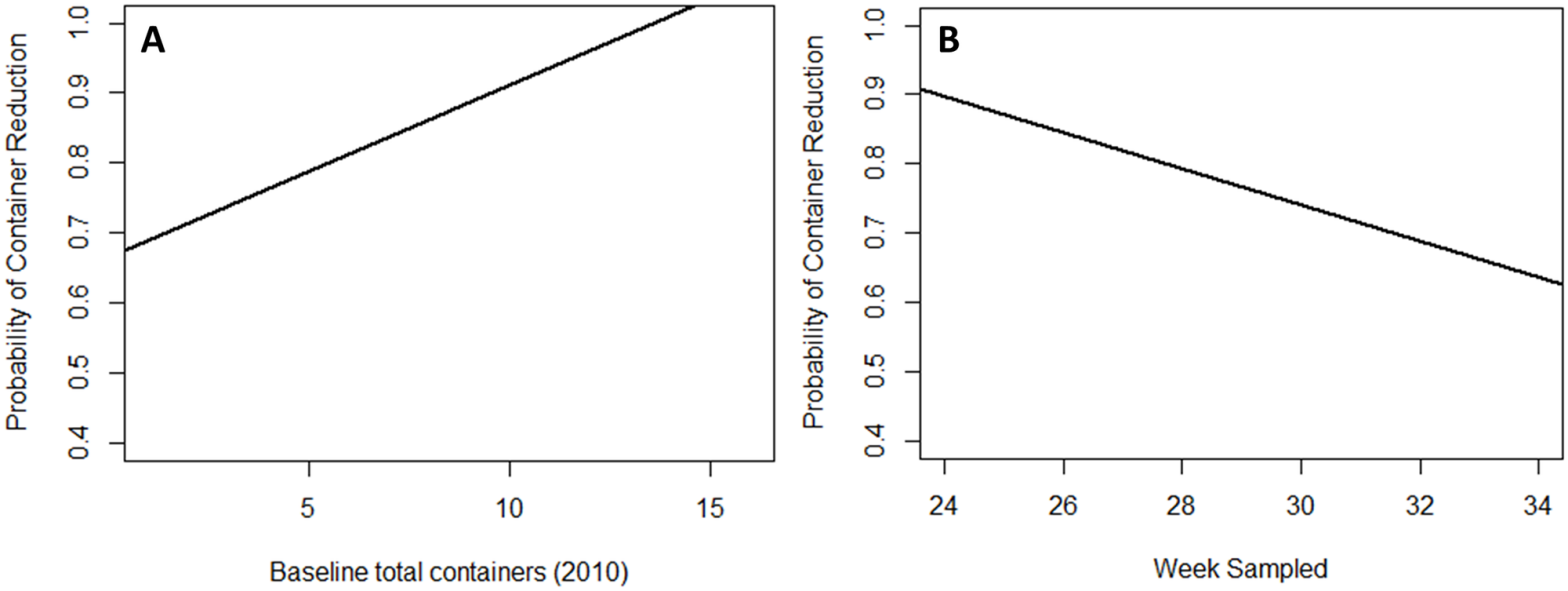
Greater container reductions in households with higher baseline containers and household sampled early season. Relationship between (A) numbers of baseline (2010) water-holding containers and (B) sampling week with probability of container reduction from 2010 to 2011.

Abundances of total and mosquito-infested containers were not significantly different among households that received education materials, control households, and households that had not been previously visited (double control) in 2011 (Shepherd Park; p = 0.944) and 2012 (Silver Spring; p = 0.642). Container reduction from 2010 to 2012 was predicted by an interaction between baseline container number in 2010 and education intervention (Table 2), with greater probability of container reduction in control households than households that received education materials, particularly when households had low numbers of baseline (2010) containers (OR = 4.88; Fig 2). Container reduction from 2010 to 2011 was predicted by baseline container numbers and week (Table 2). Households were more likely to have reduced container numbers if they independently had higher numbers of baseline (2010) water-holding containers (OR = 1.40) or were sampled early in the season (OR = 1.20; Fig 3). Results of Fisher’s Exact Test indicated a lack of association between self-reported source reduction practice adoption (OR = 0.256; p = 0.106) and actual container reduction from 2010 to 2012.

**Table 2:** Logistic regression results testing the effects of education intervention on household-level reductions of total containers in 2011 and 2012, compared to 2010.

	2011			2012		
Factors ^a^	df	X^2^	p ^b^	df	X^2^	p ^b^
Education	1, 192	0.88	0.350	1, 131	0.00	0.999
Neighborhood	5, 192	2.92	0.710	5, 131	1.33	0.932
Sample Week	1, 192	15.79	**< 0.001**	1, 131	2.62	0.106
Baseline Containers	1, 192	13.52	**< 0.001**	1, 131	2.79	0.095
Neighborhood X Education	-	-	-	5, 131	1.81	0.875
Week X Education	-	-	-	1, 131	1.11	0.292
Baseline Containers X Education	-	-	-	1, 131	4.95	**0.026**

^a^ Neighborhood, sample week, and baseline (2010) containers were included in all models. Interactions were significant in 2012and therefore shown.

^b^ Effects significant at experimentwise α = 0.10 are shown in bold.

**Fig 4:**
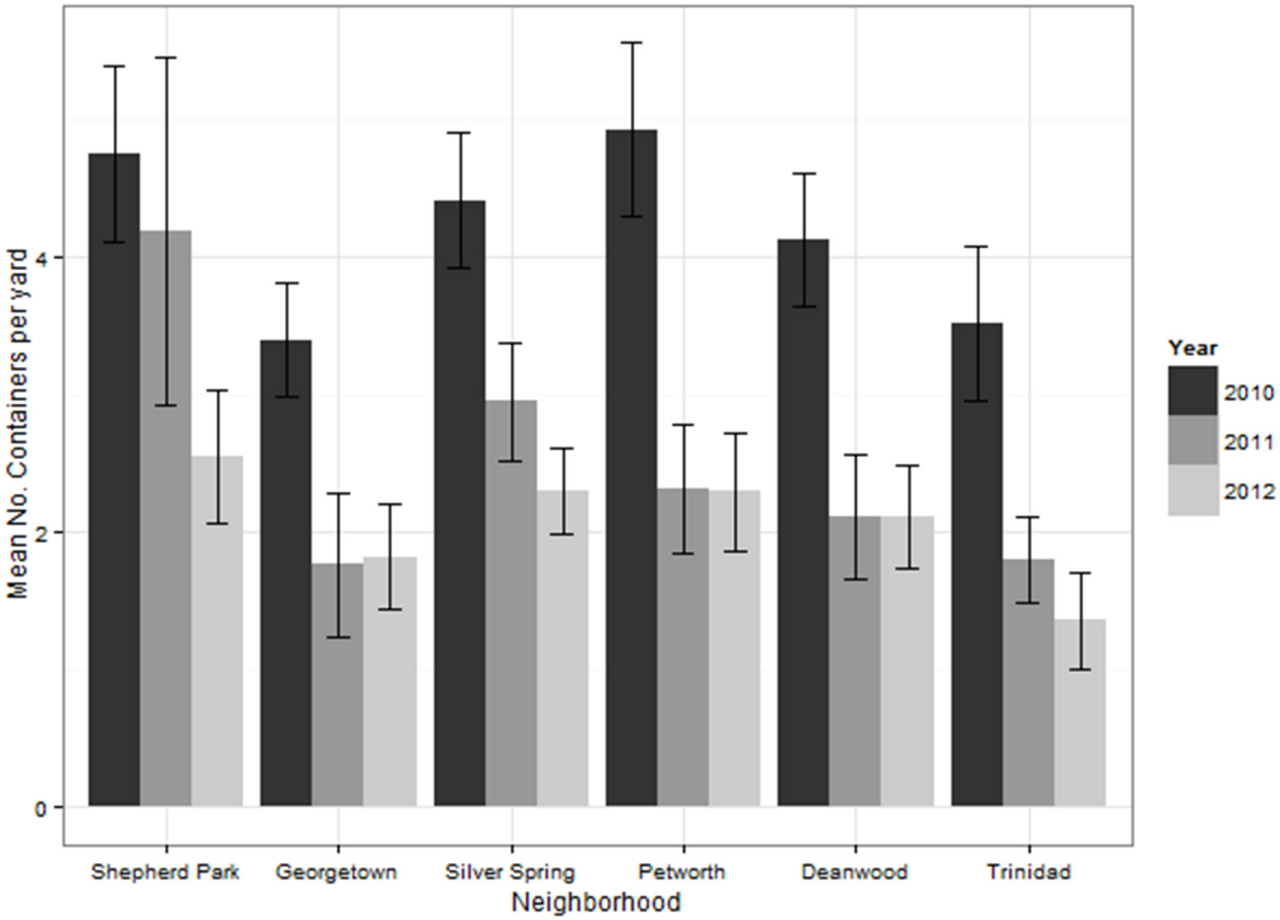
Container numbers decrease each year. Mean number of water-holding containers per household yard in each neighborhood in 2010, 2011, and 2012.

**Fig 5:**
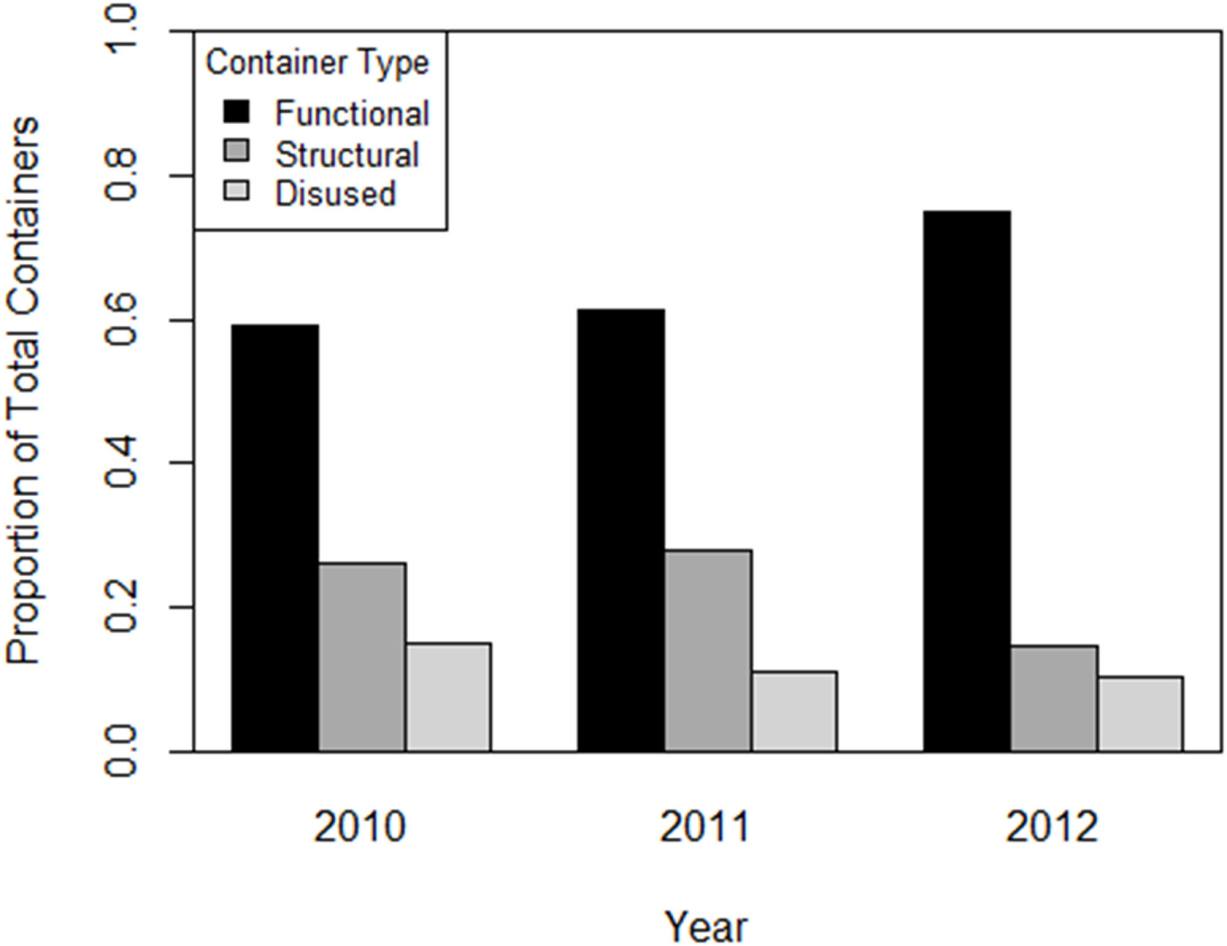
Proportions of each container type varied with sample year. Proportion of water-holding containers by container type per household yard in 2010, 2011, and 2012. See text for container definitions.

There was a 45.4% and 67.6% decline in the number of water-holding containers surveyed in 2010 (n = 1,012) with 2011 (n = 552), and with 2012 (n = 328), respectively (Fig 4). Functional containers accounted for 62.3% (1,179/1,892) of total sampled containers, increasing from 58.8% (595/1012) in 2010 to 75.0% (246/328) in 2012 (Fig 5). Household reductions of functional containers was predicted by baseline (2010) container numbers in both years, with a greater likelihood of functional container reduction in households that had more baseline (2010) containers (2011: OR = 1.22; 2012: OR = 1.53) (Table 3). Reductions of structural containers were predicted by sampling week (p = 0.019) and education intervention (p = 0.098; Table 3) in 2012. The probability of structural container reduction in 2012 was greater if the household was sampled earlier in the season (OR = 0.82) and lower in households that received education materials (OR = 0.42; Fig 5). Disused container reduction was predicted by baseline containers in 2011 (OR = 1.27; Table 3) only, with increased probability of disused container reduction in households that had more baseline (2010) disused containers.

**Table 3:** Logistic regression results testing the effects of education intervention on household reductions of structural, functional, and disused containers from 2010 to 2011 and from 2010 to 2012.

	2011			2012		
	df	X^2^	p ^a^	df	X^2^	p ^a^
**Functional Containers**						
Education	1, 165	0.35	0.554	1, 105	0.51	0.476
Neighborhood	5, 165	6.07	0.299	5, 105	4.51	0.480
Week	1, 165	0.58	0.445	1, 105	0.33	0.570
Baseline Containers	1, 165	7.59	**0.006**	1, 105	13.17	**<0.001**
**Structural Containers**						
Education	1, 148	0.08	0.775	1, 94	2.73	**0.099**
Neighborhood	5, 148	6.59	0.252	5, 94	4.32	0.504
Week	1, 148	0.28	0.600	1, 94	5.54	**0.019**
Baseline Containers	1, 148	0.02	0.880	1, 94	1.76	0.184
**Disused Containers**						
Education	1, 64	1.07	0.300	1, 39	0.001	0.977
Neighborhood	5, 64	2.99	0.701	5, 39	1.09	0.955
Week	1, 64	0.27	0.604	1, 39	1.57	0.211
Baseline Containers	1, 64	3.33	**0.068**	1, 39	0.63	0.428

See text for container definitions.

^a^ Effects significant at experimentwise α = 0.10 are shown in bold.

A total of 15,604 immature mosquitoes (larvae + pupae) were collected in 2011 and 2012 of this study, to complement the 24,375 mosquitoes collected by Dowling et al. in 2010 [31]. The two most common species over the entire three years were *Ae*. *albopictus* and *Cx*. *pipiens*, which accounted for 51.7% (n = 17,727) and 32.8% (n = 11,238) of total larvae (n = 34,308), respectively. The remaining larvae consisted of *Culex restuans* Theobald (5.2%), *Aedes triseriatus* Say (3.3%), *Aedes japonicus* Theobald (7.0%), and *Toxorhynchites* species (<1.0%). From 2010 to 2012, the proportion of mosquito-positive containers across all households increased from 30.3% (307/1,012) to 47.8% (157/331). Change in the proportion of mosquito-positive containers was consistent with change in *Ae*. *albopictus*, which increased from 51.4% (10,947/21,283) to 65.4% (7,930/9,204) of total larvae, but not those of *Cx*. *pipiens*, which decreased from 37.3% (8,669/21,283) to 17.1% (1,577/9,204).

Overall, reductions in containers were associated with decreases in mosquito abundances, particularly those of *Ae*. *albopictus* (Table 4). This association appeared to be mostly driven by reductions in functional and structural containers. Reductions of functional containers had significant associations with decreases in all mosquito variables in 2011 and almost all variables (except *Cx*. *pipiens* larvae) in 2012. Reductions in structural containers were associated with reductions of total immatures and *Cx*. *pipiens* larvae in 2011 but with total immatures, total pupae, *Ae*. *albopictus* larvae and *Ae*. *albopictus* pupae in (Table 4). Reductions of disused containers were only associated with the reduction of total pupae in 2012, and no mosquito variables in 2011 (Table 4).

**Table 4:** Results of Fisher’s exact tests of associations (p-values) between container reduction and decreased estimated mosquito abundance for household from 2010 to 2011 and 2012.

	Total Immatures	Total Pupae	*Ae*. *albopictus* Larvae	*Ae*. *albopictus* Pupae	*Cx*. *pipiens* Larvae	*Cx*. *pipiens* Pupae
**2011**						
**Total**	**<0.001 (7.41)**	0.101	**<0.001(7.41)**	**0.040(2.99)**	**0.025(3.13)**	0.213
**Functional**	**<0.001(11.53)**	**0.008(4.37)**	**<0.001(10.19)**	**0.014 (4.91)**	**0.002(6.65)**	**0.006(6.60)**
**Structural**	**0.028(3.18)**	0.554	0.394	0.395	**0.057(2.76)**	0.395
**Disused**	0.519	0.352	0.340	0.715	0.520	0.649
**2012**						
**Total**	**0.001(5.51)**	**0.074(2.50)**	**0.002(4.90)**	**0.086(2.39)**	0.241	0.167
**Functional**	**0.003(3.54)**	**0.016(2.97)**	**0.005(3.44)**	**0.035(2.50)**	**0.099(1.82)**	0.152
**Structural**	**0.006(3.68)**	**0.018(3.27)**	**0.038(2.64)**	**0.026 (3.01)**	0.650	0.669
**Disused**	0.521	**0.084(7.61)**	0.407	**0.084(7.61)**	0.621	0.521

Significant results at experimentwise α = 0.10 are shown in bold and have Odds Ratios reported in parentheses.

## Discussion

In this study, print education materials appear to have little effect at reducing mosquito container habitat in residential yards. Although the number of water-holding containers declined by 67.6% from 2010 to 2012, it is unlikely that this decline was due to our education intervention because we detected greater container reductions in control households, indicating a negative influence of our intervention materials on household source reduction practices. These findings are broadly similar with those of other studies in the United States, in New Jersey [22] and Florida [25], which also did not find significant container reductions associated with a print education intervention. Collectively these findings are consistent with the idea that print education campaigns are insufficient to reliably motivate resident-based mosquito habitat reduction [22, 25, 42–43], and may even have the unintended effects of increasing mosquito habitat and decreasing resident concern for vector species.

Our study showed decreases in overall mosquito abundances with reductions of container habitat, indicating that resident-based mosquito management can be effective at reducing the development of vector mosquitoes and exposure to biting adults. Household container reductions were more closely associated with decreases in *Ae*. *albopictus* than *Cx*. *pipiens*. However, despite overall decreases in container habitat and *Ae*. *albopictus*, the proportion of total mosquito-positive containers increased over the study duration, and this increase was mostly due to *Ae*. *albopictus* activity. Past work has shown that *Ae*. *albopictus* tends to opportunistically select oviposition sites [42, 44] and utilizes a broad range of container types and sizes, including small ephemeral containers that may harbor small populations of this invasive species [45, 46]. In contrast, *Cx*. *pipiens* tends to prefer larger containers [46]. Residents appear to be able to decrease abundances of developing *Ae*. *albopictus*, and likely their exposure to biting *Ae*. *albopictus* adults, by reducing small containers that may be relatively easier to manage than larger containers. Residents appear to manage some containers that are utilized by *Cx*. *pipiens* and thus reduce the proportion of total containers with this species. However, there are likely a number of larger containers that harbor many *Cx*. *pipiens*, which are more difficult to permanently eliminate (e.g., children’s toys, disconnected downspouts, fish ponds).

Our findings relating reductions of individual container types with decreases in *Ae*. *albopictus* and *Cx*. *pipiens* may further reflect species-specific oviposition preferences. We found significant associations between decreases in *Cx*. *pipiens* larvae with reductions of functional containers in both 2011 and 2012, and structural containers in 2011. Past studies have shown that structural and functional containers have larger average volumes [e.g., 32, 42]. Coupled with the fact that these containers are likely to be regularly used, our results are consistent with the idea that if residents can manage these containers (and consequently decrease *Cx*. *pipiens* abundances) they would most likely do it over the first year of the study (i.e., 2011). We observed that decreases in disused containers, which are on average smaller than functional and structural containers [32], were associated with reductions of total and *Ae*. *albopictus* pupae in 2012 but not in 2011. Disused containers may be less obvious and more difficult for residents to locate and access [27]; thus, restricting measurable decreases in their abundance to the second year of our study (i.e., 2012).

We observed substantial reductions in mean container numbers from 2010 to 2012. This finding might have been partly due to an unintended, indirect, influence of investigator visits that was irrespective of any education. However, we detected no differences in container numbers between newly sampled households, which had not previously been visited, with control and experimental households. An alternate and more likely explanation for our findings is related to rainfall. In our study period, there were considerable decreases in mean summer (June-August) precipitation between 2010 and 2012 (966 mm to 736 mm) (NOAA, Baltimore City station, http://www.ncdc.noaa.gov/), which almost certainly reduced numbers of containers that were holding water. Lower rainfall and decreased numbers of water-holding containers may be expected to affect *Cx*. *pipiens* more than *Ae*. *albopictus*. *Aedes albopictus* oviposit desiccation-resistant eggs that hatch when flooded, whereas *Cx*. *pipiens* oviposit egg rafts that hatch within a few days. Since ovipositing *Cx*. *pipiens* require existing aquatic habitats, we would expect them to be more strongly limited by their availability and experience reductions in abundances sooner, such as in the first year of our study, than *Ae*. *albopictus*. *Aedes albopictus* has been shown to recolonize containers within a few weeks despite source reduction [42]. These life history traits may allow *Ae*. *albopictus* to utilize temporary disused containers, which may have helped *Ae*. *albopictus* increase its regional dominance from 2010 to 2012 in this study.

This study found that residents with higher knowledge and in households that received education materials were more likely to report that they adopted source reduction. These findings suggest that the education materials may promote mosquito management by individuals independent of any changes in their knowledge or attitude. However, individual source reduction adoption was not significantly associated with household container reduction, a finding that is similar to other studies that have shown no association between self-reported source reduction and reductions in water-holding containers [27, 30–31, 47]. One explanation for this result may be that source reduction practices that do not consist of container reduction per se (e.g., applications of mosquitocides or oils, emptying water) are the predominant form of resident-based container control. Another, possibly more likely, explanation is that individual source reduction behavior may be offset by the addition of containers from household activities or gardening practices, and may not occur following each rain event.

Education intervention was not related to changes in individual knowledge but was related to concern of mosquito transmitted diseases. Unexpectantly, respondents from households that received education materials showed a greater decrease in concern than respondents from control households. This result may be due to an increased awareness of specific mosquito-borne illnesses from the education materials and a perception that they do not pose as great a health risk is previous thought. Anecdotally, we noticed that when some residents understood that the most important mosquito-borne threat in the region is West Nile virus as opposed to other diseases with greater negative media attention and public health impacts, including as HIV or Ebola, they appeared less concerned of mosquito vectors. Collectively, these findings suggest that not only were our print education materials ineffective at increasing household level mosquito management, it also had limited and unexpected impact on the knowledge and attitudes of individual residents. Although we administered education materials to intervention households, we cannot ensure that all or even some of the residents read them. Surveys are inherently limited in their ability in collect valid data on social factors and processes, including a resident's recollection of earlier events, such as the amount of time reading materials that were administered the prior year or at the start of a summer season. Other social science instruments, including focus groups and interviews, may be needed to more thoroughly assess resident comprehension of materials and other education approaches in future work.

WNV incidence has been associated with significant public health implications, including exacerbating symptoms in immunocompromised and elderly residents [4]. Existing lay knowledge of local arboviruses should be incorporated when designing education campaigns and community outreach programs to address pre-existing assumptions that may inhibit resident source reduction behaviors [43], especially given the rising threat of arbovirus invasion as climate change makes previously unavailable ranges more amenable to invasive pest species [11, 15, 48]. Print education materials that have been designed in partnership with target communities have been shown to be effective in other mosquito control studies, serving the dual purpose of intensively educating a subset of the community that help design the materials, as well as tailoring the message to incorporate the social, cultural, and environmental factors of the wider target population [28, 30].

Findings from this study are similar to the conclusions of other studies [19, 23–24, 26, 29], which are increasingly endorsing multifaceted approaches to mosquito control, consistent with integrated mosquito management principles. Human-mosquito systems are an important model for developing new socio-ecological theory for human-pest interactions, as well as engaging community participation in the broader goals of improving urban quality of life and neighborhood revitalization. Passive education material should be disseminated in conjunction with active community engagement, and should be tailored to the specific social, economic, and ecological characteristics of the target area [27–28, 30]. Knowledge and awareness of mosquitoes may be insufficient to influence routine reduction of water-holding containers by residents [21, 49]. Successful mosquito reduction has been observed in studies that engage the target community through community meetings, educational training sessions, elementary school curriculums, and neighborhood clean-up events [20, 23, 28, 30]. Active education campaigns have been more effective than passive print materials alone at increasing resident knowledge of disease vectors [23], reduction of water-holding containers [30], and adult mosquito abundances in urban areas [19]. Container control strategies are increasingly targeting residences that support high levels of infestation [42], and specifically containers that support high levels of *Ae*. *albopictus* productivity [44, 50]. Future efforts to educate urban neighborhoods may be more effective if overarching sanitation and pest control issues are addressed, while incorporating community-wide active education outreaches, with passive materials designed and distributed by community members [24, 51].

## Supporting Information

S1 Fig Education Materials used in the study. The brochure (facing and reverse pages), Calendar (cover page only), notepad and magnet were sent to half of all study households in 2011 and 2012. Provided in adherence to the PLOS policy to make all data underlying the findings described in this manuscript fully available.
(DOCX)

S1 Table Raw data of the social and mosquito data. Provided in adherence to the PLOS policy to make all data underlying the findings described in this manuscript fully available.
(XLSX)
