# Supplementary figures and images for: Effectiveness of Print Education at Reducing Urban Mosquito Infestation through Improved Resident-Based Management
Source: PLoS One. 2016 May 12;11(5):e0155011. doi: 10.1371/journal.pone.0155011 (PMC4865130; doi:10.1371/journal.pone.0155011)

Brochure facing page
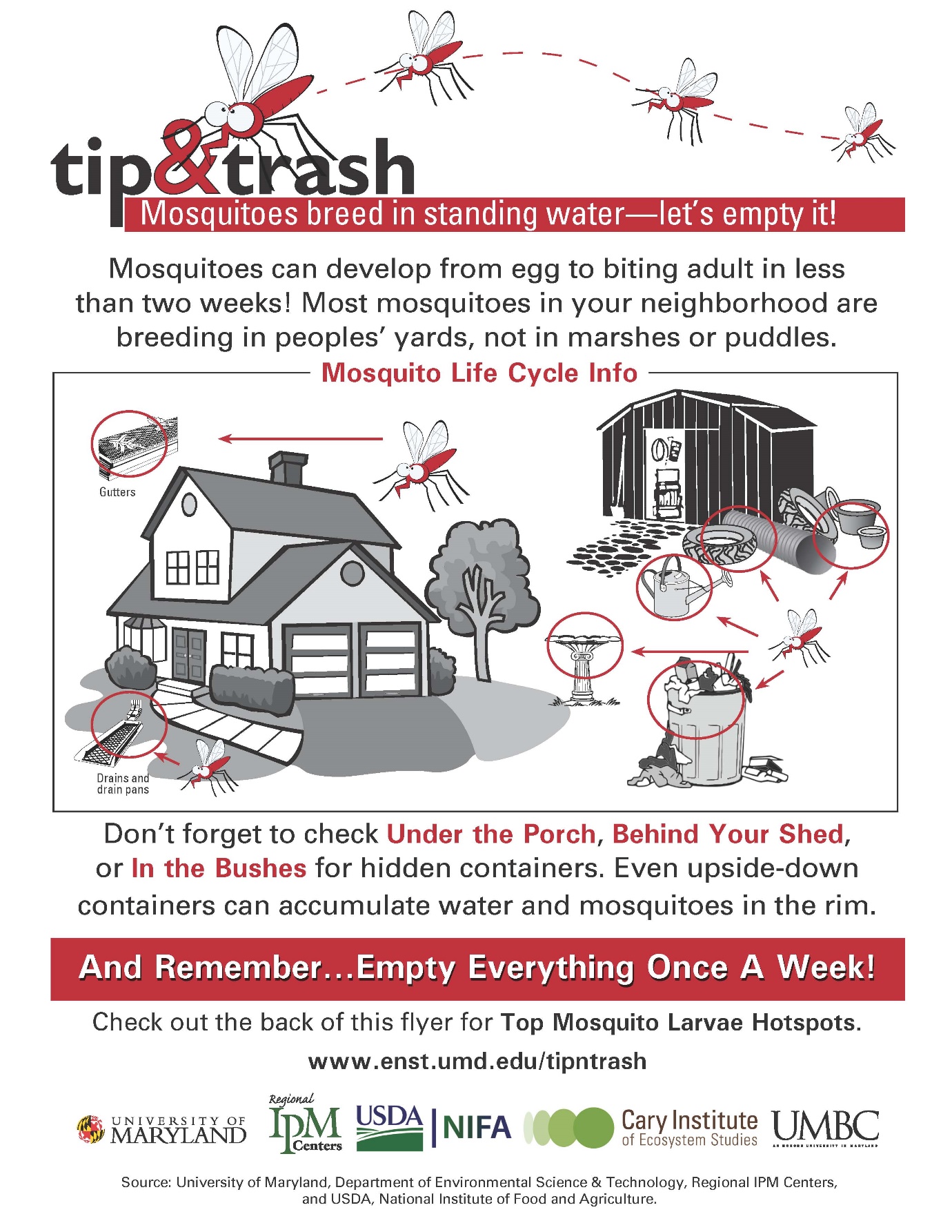


Reverse page of brochure


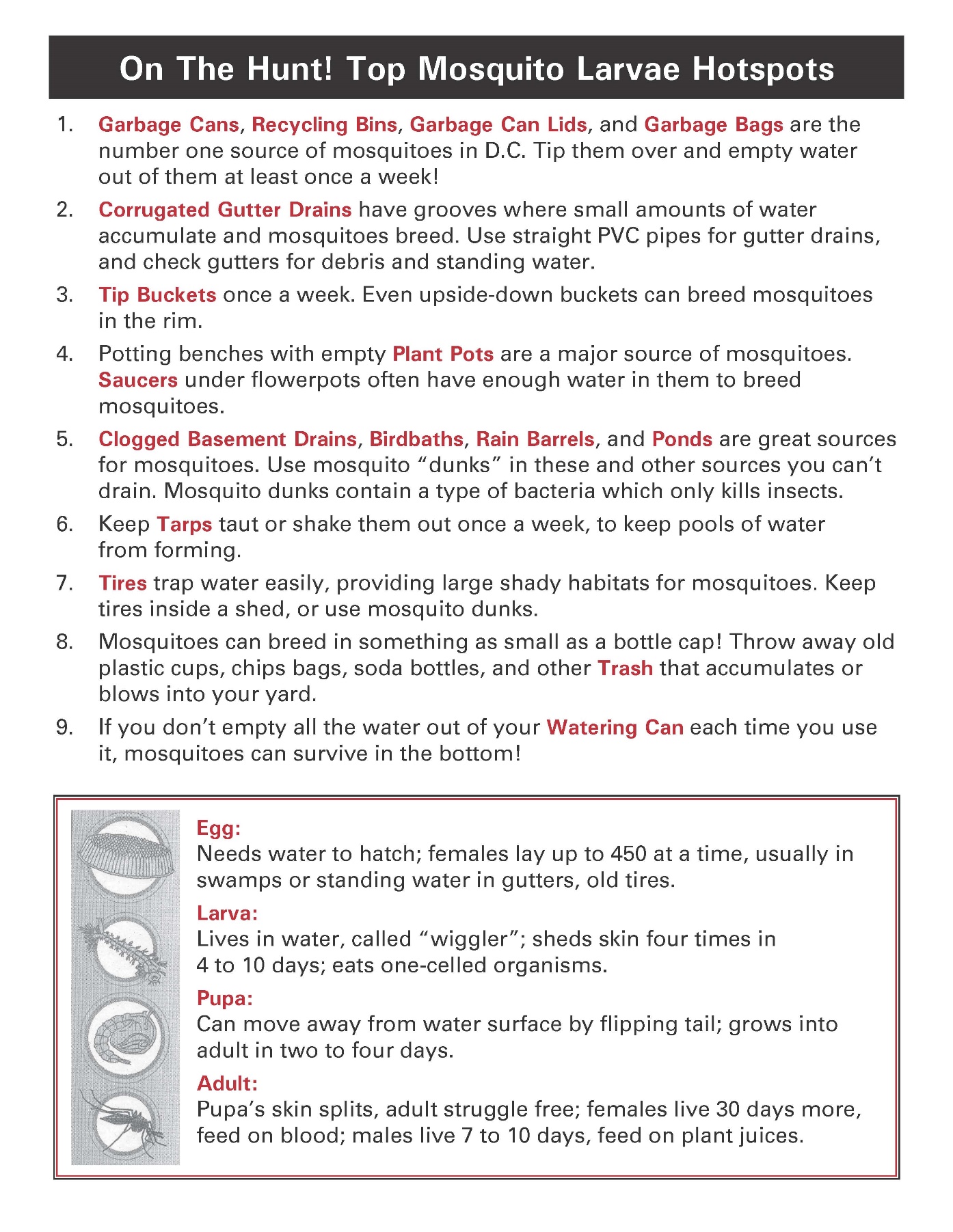


Cover page of Calendar


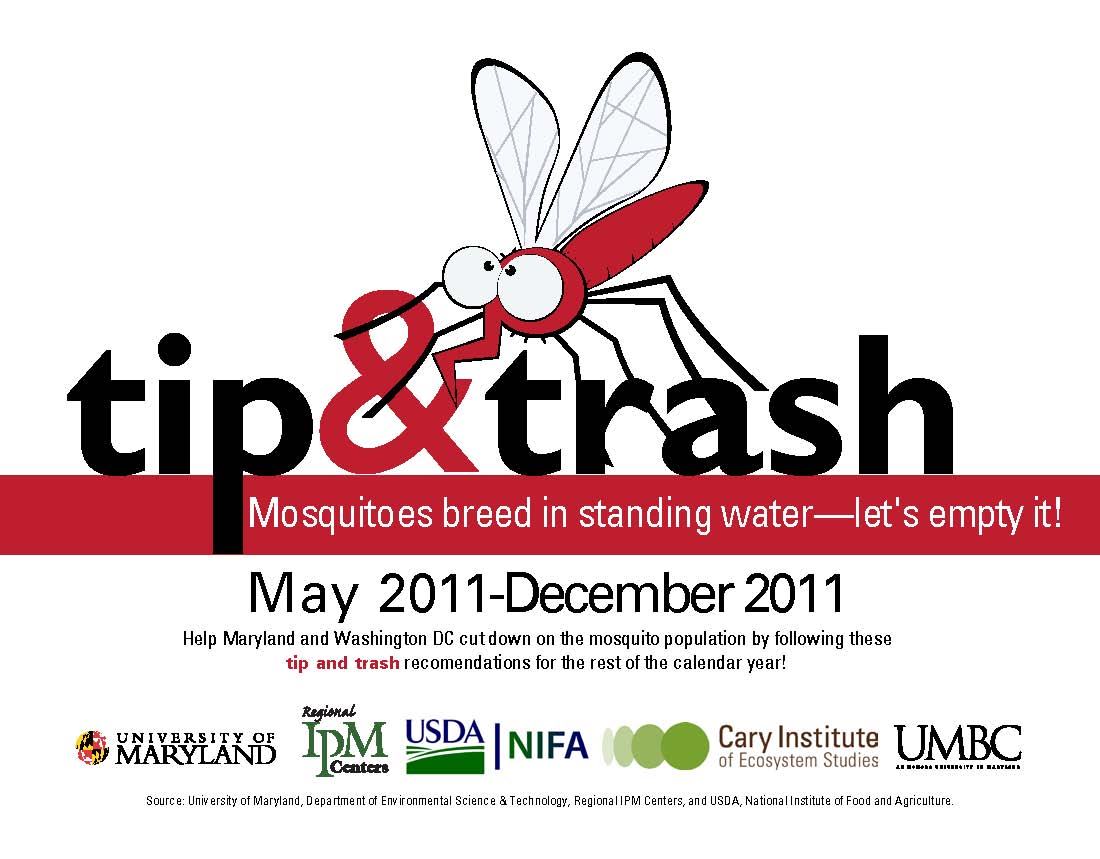


Notepad


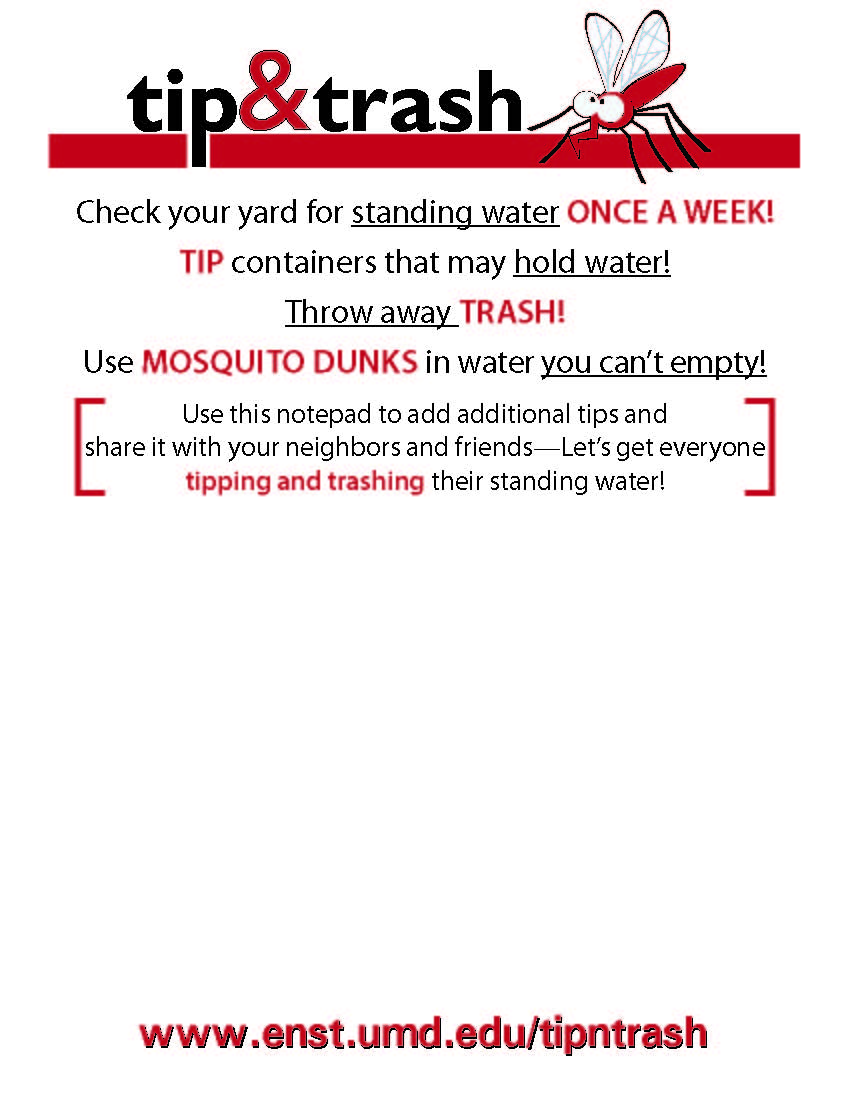


Magnet


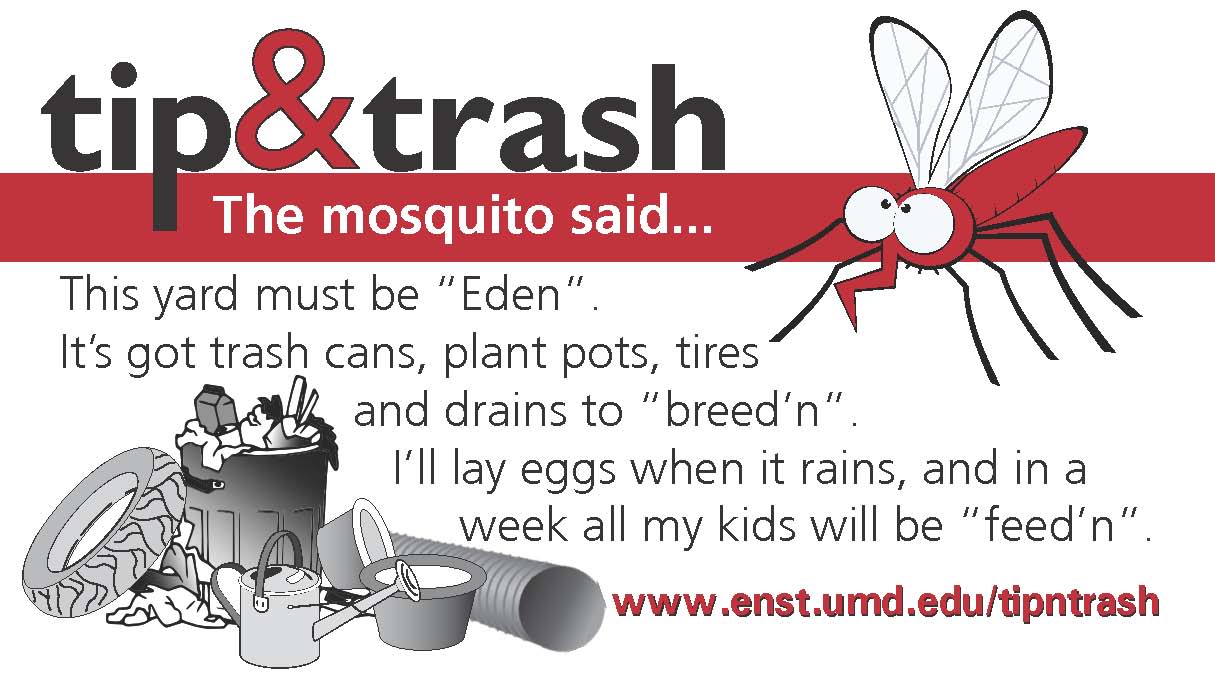

Supplement: S1 Fig — The brochure (facing and reverse pages), Calendar (cover page only), notepad and magnet were sent to half of all study households in 2011 and 2012. Provided in adherence to the PLOS policy to make all data underlying the findings described in this manuscript fully available. (DOCX) [file pone.0155011.s001.docx]
